# The Transactions of the Kentucky State Medical Society

**Published:** 1853-04

**Authors:** 


### EDITORIAL.

*The Transactions of the Kentucky State Medical Society:* at their meeting held in Louisville, on the third Wednesday of October, 1852.

This is a volume of about 350 large 8vo pages, and besides the Minutes contains several very able Reports from Committees appointed at the previous meeting. The session continued three days, and appears to have been characterized by much interest and unanimity. A large number of members were elected, and the following formidable list of Committees appointed to report at the next meeting. Standing Committees—1st, Of Arrangements; 2d, On Practical Medicine; 3d, On Improvements in Pharmacy; 4th, On Vital Statistics; 5th, On Obstetrics; 6th, On Medical Ethics; 7th, On Public Hygiene; 8th, On Epidemics; 9th, On Surgery; 10th, On Indigenous Botany, and 11th, On Finance. And the following Special Committees—1, On Medical Biography, or the Lives of meritorious or distinguished Physicians or Surgeons of Kentucky; 2, On Medical Literature, or the History of the Medical Authorship of Kentucky; 3, On the relation between Diseases and particular Geological Formations; 4, On the Statistics of Hernia; 5, On the Statistics of Lithotomy and Calculous Diseases; 6, On the History and Mode of Management of Hospitals; 7, On the History and Mode of Management of Penitentiaries and Prisons; 8, On Suits for Mal-Practice; 9, On the Results of Surgical Operations in Malignant Diseases; 10, On Epidemic Erysipelas; 11, On Epidemic Dysentery; 12, On Typhoid Fever; 13, On Placenta Prœvia; 14, On the Statistics of Remedies in Disease.

Of the papers read before the Society, the Address of the President stands first in order. It is a plain, practical paper, and is devoted to two general topics, viz:—" 1st, The establishment and maintenance of union, harmony and good government among its members, thereby promoting the character, interests, honor and usefulness of the profession. 2d, The cultivation and advancement of Medical Science and Literature, by the collection, diffusion, interchange, preservation and general circulation of medical knowledge throughout the State." Under the first division of the subject, the President indicates, what he conceives to be the reasons why the medical profession does not command the confidence and respect, which it formerly did—such as the low standard of attainments frequently possessed by those who aspire to its honors, and the great facility of gaining admission into the ranks of the profession. He justly *urges* upon the members of the profession the importance of requiring a higher standard of qualifications of students, and upon Medical Colleges of raising the standard of attainments of candidates for the Degree in Medicine. And admonishes the members of the Society to "keep up with the times themselves," by reading the medical literature of the day, and by contributing their own experience and observations to the medical public, by *writing* for the journals. And not the least important part of the Address, (if practicable) is that which suggests the importance of devising some plan of general application, by which medical practioners may be relieved from the onerous duty of giving their services, medicine, and frequently food and raiment, with no prospect of remuneration, to the pauper classes, always large in every community.

The next paper is a Report by Doctor Chipley upon Vital Statistics. It is more a historic glance of the subject than a critical analysis; it contains, however, some valuable suggestions, and is written in a forcible and elegant style. An interesting feature of the Report is the accompanying map of Kentucky, "so colored as to represent, at a single glance, the relative mortality of the different portions of the State."

The Report on Surgery by Dr. S. D. Gross, is a valuable contribution to the medical literature of Kentucky; it evinces great industry and patient research, and is indeed a very complete history of surgery in that State, commencing with the earliest times and extending to the present, it occupies near 200 pages of the volume. In the report Dr. Gross gives a very interesting Memoir of one of Kentucky's first and ablest Surgeons, Dr. Ephraim McDowell, for whom he claims the credit of first performing ovaritomy, which was done as early as 1809, and was perfectly successful. It is not certain how many operations of this kind Dr. McD. performed, but it is supposed that he made the ovarian section thirteen times. Of the first six operations, but one proved fatal. In this paper Dr. G. notices most of the operations in Surgery of every kind, that have been published, making it really little short of a treatise upon the subject.

The Report on Indigenous Botany is a well written article, containing some practical suggestions upon the effects of vegetables upon the animal economy, and the importance of directing more attention to this class of remedies. It gives a description of a few individual plants, and a list of the plants of Kentucky.

Anything that will induce the medical practitioner to *observe* more closely, and *record* what passes in his practice, must be of value both to himself and his patient. The more accurately he observes, the more likely will he be to arrive at a correct diagnosis, upon which depends success in practice. Therefore we are pleased with the recommendation of the Committee on "Case Book" which is to be used, not in recording the extraordinary cases only, but *all* cases coming under his care; the plan suggested is simple, and must lead to greater system in examinations and more care in diagnosis.

Reports were also made upon Medical Ethics and Obstetrics, and papers were read upon Epidemics.

It is gratifying to witness the increasing interest manifested in different sections of the country, in the elevation and improvement of the medical profession. State organizations like that of Kentucky, must exert a salutary influence upon the profession, and will interest every member who properly estimates its usefulness and honor.

The volume gives good evidence of the industry, zeal and ability of the profession in Kentucky; we say to our brethren in other States, "go and do likewise."

